# Dataset of sentiment tagged language resources for Macedonian language

**DOI:** 10.1016/j.dib.2025.112384

**Published:** 2025-12-12

**Authors:** Sofija Kochovska, Jernej Vičič, Branko Kavšek

**Affiliations:** University of Primorska, FAMNIT, Glagoljaska 8, 6000 Koper, Slovenia

**Keywords:** Macedonian language, Sentiment analysis, Sentiment lexicon, Rule-based methods, Natural language processing, Low-resource languages, AnA words, Intensifiers

## Abstract

Macedonian is a South Slavic language spoken by about 2 million people, primarily in North Macedonia and among diaspora communities worldwide. It’s known for a few distinctive features. Most notably, it uses definite articles attached to the end of nouns, for example, *kniga* (a book) becomes *knigata* (the book). Furthermore, it doesn’t use grammatical cases, which makes its grammar relatively straightforward compared to other Slavic languages.

The dataset comprises two lists of sentiment annotated words that present the core of the Macedonian sentiment-annotated lexicon, a list of the stopwords, and a list of Affirmative and non-Affirmative words (AnAwords) composed mostly of intensifiers and diminishers, and a list of polarity shifters. The main usage of the presented materials is in rule-based sentiment analysis, but the usage of some of the lists can be much broader.

Specifications Table

**Table:** 

Subject	Computer Science Applications
Specific subject area	Natural language processing
Type of data	Analyzed, raw
Data collection	For the lexicon, we utilized the Bosnian sentiment annotated lexicon [1], which contains 3935 negative lemmas and *1219* positive lemmas which were all translated into Macedonian and hand-checked for possible changes of sentiment. The auxiliary lists (stopwords, AnAwords, and the list of polarity shifters) were loosely based on the Bosnian dataset [4], but heavily modified to account for some of the differencies between the source and target language. The lists are by no means exhaustive. An extended description of the creation process is presented in Section EXPERIMENTAL DESIGN, MATERIALS AND METHOD.
Data source location	Primary data source (partial): Bosnian sentiment annotated lexicon presented in Jahić and Vičič [1]. Data source location: University of Primorska, UPFAMNIT, Glagoljaska 8, 6000 Koper, Slovenia
Data accessibility	***Sentiment polarity lexicon of Macedonian language*** [3] Repository: Zenodo DOI: 10.5281/zenodo.15790588 URL: https://doi.org/10.5281/zenodo.15790588 ***Lists of stopwords, polarity shifters and AnAwords of Macedonian language*** [2] Repository: Zenodo DOI: 10.5281/zenodo.15794763 URL: https://doi.org/10.5281/zenodo.15794763
Related research article	*None*

## 1 Value of the Data

The presented dataset of sentiment analysis resources for Macedonian language is composed of a lexicon of sentiment tagged words, a list of Affirmative and non-Affirmative words (AnAwords), a list of stopwords, and a list of polarity shifters. It holds significant value for a range of applicable and research projects. All steps of translation, manual review, and validation are described to enable replication for other South Slavic languages.

• The main potential users of the data are researchers and developers in the scientific subfield of natural language processing (NLP), the field of sentiment analysis, who benefit from all the enclosed resources when using rule-based methodology. Developers of information retrieval systems gain from stopword removal, as it simplifies the retrieval process and enhances both efficiency and accuracy. Likewise, creators of online search engines and catalogs profit from this approach, since eliminating stopwords results in faster and more concise searches.
• The data can be easily reused by other researchers through the stopwords list, which functions as a lookup dictionary for quick identification and removal of stopwords from text. Meanwhile, the AnAwords list is essential for detecting amplifiers that strengthen the meaning of nearby words. The sentiment lexicon provides a structured basis for recognizing sentiment-labeled words. By applying this lexicon, developers and researchers can more effectively identify and separate sentiment-bearing terms from neutral ones, thereby improving the quality of sentiment analysis.

## 2 Background

Macedonian can be occasionally confused with Bulgarian because they share some similarities in vocabulary and grammar. However, the two languages followed different historical and linguistic paths. Bulgarian developed more under Ottoman and Russian influence, while Macedonian was standardized in the 20th century based on local dialects from central and western parts of the country. The result is a language with its own literary tradition, phonetic system, and evolving identity.

Though closely related, Macedonian stands as a distinct language which is shaped by its speakers, geography, and history.

The primary objective of this study is to introduce a set of valuable resources for sentiment analysis in the Macedonian language, comprising a comprehensive sentiment lexicon, an AnAwords list, a polarity shifters list, and a stopwords list. These resources are intended to support researchers and developers engaged in Natural Language Processing (NLP) tasks, as well as applications such as online search engines and information retrieval systems. Although contemporary research in NLP is predominantly driven by deep learning and statistical approaches, there remains a substantial need for carefully constructed linguistic resources, particularly for under-resourced languages.

Macedonian is an under-resourced language and to the author’s knowledge, the only published research in the field of sentiment analysis is by Jovanoski et al. [6] that produced an automatically tagged lexicon. A python script has been added to our dataset that merges both lexicons.

## 3 Data Description

The dataset is built for the Macedonian language, which uses the Cyrillic script. This script consists of 31 letters and is highly phonemic, therefore each sound corresponds to a single letter. This feature simplifies text processing tasks such as tokenization, normalization, and lemmatization, making it well-suited for rule-based sentiment analysis.

The dataset consists of six UTF-8 encoded plain-text files that form a sentiment-annotated lexicon and accompanying resources for the Macedonian language. Each file contains one entry per line, now including both the Macedonian word and its English translation.

1. **MK_POSITIVE.txt**
  ○ **Entries**: 1204
  ○ **Content**: Macedonian words expressing positive sentiment, alongside English translations
  ○ **Format**: One lowercase word per line, in Cyrillic script, followed by a comma and its English translation
  ○ **Use**: Increases sentiment score during classification

The positive sentiment words are words that in most cases reflect a positive sentiment when used in a text.

2. **MK_NEGATIVE.txt**
  ○ **Entries**: 2341
  ○ **Content**: **Macedonian** words associated with negative sentiment, alongside English translations
  ○ **Format**: One lowercase word per line, in Cyrillic, followed by a comma and its English translation
  ○ **Use**: Decreases sentiment score during classification

The negative sentiment words are words that in most cases reflect a negative sentiment when used in a text.

3. **MK_AnAwords_intensifiers.txt**
  ○ **Entries**: 69
  ○ **Content**: Macedonian intensifiers (words that amplify sentiment), alongside English translations
  ○ **Format**: One lowercase word per line, in Cyrillic script, followed by a comma and its English translation
  ○ **Use**: Multiplies the sentiment strength of nearby words

Intensifiers are words or expressions in a text that increase the strength, degree, or emphasis of another word.

4. **MK_AnAwords_diminishers.txt**
  ○ Entries: 37
  ○ **Content**: Macedonian diminishers (words that reduce sentiment intensity), alongside English translations
  ○ **Format**: One lowercase word per line, in Cyrillic script, followed by a comma and its English translation
  ○ **Use**: Lowers the sentiment strength of nearby words

Diminishers (sometimes called downtoners or hedges) are the opposite of intensifiers. Instead of amplifying meaning, they weaken, reduce, or soften the strength of the word they modify.

5. **MK_AnAwords_polarityShifters.txt**
  ○ **Entries**: 21
  ○ **Content**: Macedonian polarity shifters (negations and reversers), alongside English translations
  ○ **Format**: One lowercase word per line, in Cyrillic script, followed by a comma and its English translation
  ○ **Use**: Inverts the polarity of sentiment-bearing words

Polarity shifters are words or expressions that change the polarity (positive –> negative) or intensity of sentiment-bearing words in a text.

6. **MK_AnAwords_stopwords.txt**
  ○ **Entries**: 311
  ○ **Content**: Common Macedonian stopwords (e.g., prepositions, conjunctions, pronouns), alongside English translations
  ○ **Format**: One lowercase word per line, in Cyrillic script, followed by a comma and its English translation
  ○ **Use**: Filtered out during preprocessing to reduce noise in analysis

Stopwords are very common words in a language that usually carry little or no meaningful content for tasks like sentiment analysis, information retrieval, or text mining.

## 4 Experimental Design, Materials and Method

The development of the Macedonian sentiment lexicon and associated wordlists began with an initial translation of a Bosnian sentiment lexicon as the foundational resource. This base was carefully adapted to fit the linguistic and cultural nuances of the Macedonian language through extensive manual revision, ensuring both accuracy and naturalness. The Macedonian sentiment lexicon is primarily derived from the Bosnian resource. The vast majority of entries were directly translated and manually validated by native speakers. In addition, a notable number of Macedonian-specific terms, including slang, idiomatic expressions, and culturally specific sentiment words, were added during the refinement process. These new entries ensure adequate coverage of Macedonian sentiment usage, but they represent only a small portion of the final lexicon compared to the translated core. To further validate the quality and relevance of the manually translated lexicon, the resulting entries were reviewed by a group of five native Macedonian speakers, including family members, colleagues, and academic peers. This collaborative review ensured that the lexicon accurately reflected sentiment usage in Macedonian, accounting for regional and contextual variations in meaning.

The original Bosnian lexicon was organized into six distinct categories: positive words, negative words, intensifiers, diminishers, polarity shifters, and stopwords. This classification was preserved in the Macedonian adaptation to maintain structural consistency. Following manual translation, the lexicon was enriched with additional entries relevant to Macedonian sentiment expression. All words were normalized to lowercase Cyrillic script and saved as UTF-8 encoded plain text files. Multiple rounds of thorough consistency checks were conducted to eliminate overlaps between categories and to guarantee internal coherence. Afterwards, each Macedonian entry was translated to English, to provide more context to non-Macedonian speakers. The final outcome consists of six carefully curated files, reflecting the structure of the original Bosnian lexicon, but fully refined and tailored for the Macedonian language.

The sentiment classification is rule-based and uses a scoring system influenced by modifiers. Input text is preprocessed by cleaning URLs and usernames, normalizing repeated characters, removing stopwords, and applying lemmatization via the Classla NLP pipeline [5]. Words from the positive and negative lists contribute to a cumulative score, adjusted by intensifiers, diminishers, and polarity shifters within a three-word window. Final sentiment is determined by comparing the normalized score to a threshold.

An extended version of the lexicon was created by incorporating manually scored words, including entries from Jovanoski et al. [6]. A script separates these words by polarity and appends them to the corresponding files, enabling comparisons between the initial and enriched lexicons.

## Limitations

The presented resources are manually curated and therefore not exhaustive. The coverage of contemporary language, including slang, informal expressions, and rapidly evolving vocabulary, particularly from digital and social media contexts, remains limited. Future work to address these limitations should expand the lexicon with these domain-specific and informal terms and develop more nuanced, context-aware handling of linguistic phenomena like negation and emphasis to better capture modern Macedonian usage.

## Ethics Statement

The authors have read and follow the ethical requirements for publication in Data in Brief and confirm that the current work does not involve human subjects, animal experiments, or any data collected from social media platforms.

## Credit Author Statement

• Sofija Kochovska: Conceptualization, Software, Investigation, Writing – Review & Editing, Data Curation
• Jernej Vičič: Conceptualization, Investigation, Writing – Review & Editing, Data Curation, Supervision.
• Branko Kavšek: Conceptualization, Investigation, Writing – Review & Editing, Data Curation, Supervision.

## Data Availability

ZenodoSentiment polarity lexicon of Macedonian language (Original data)
ZenodoLists of stopwords, polarity shifters and AnAwords of Macedonian language (Original data).

## References

[1] Jahić S., Vičič J. (2024). Dataset of sentiment tagged language resources for Bosnian language. Data Br..

[2] S. Kochovska, B. Kavšek, J. Vičič, Lists of Stopwords, Polarity Shifters and AnAwords of Macedonian language, 2025, Zenodo,. DOI: 10.5281/zenodo.15794763.

[3] Kochovska S., Kavšek B., Vičič J. (2025). Sentiment polarity lexicon of Macedonian language. Zenodo.

[4] Jahić S., Vičič J. (2023). Zenodo.

[5] Ljubešić N., Terčon L., Dobrovoljc K. (2024). Conference on Language Technologies and Digital Humanities (JT-DH-2024).

[6] D. Jovanoski, V. Pachovski, P. Nakov, Sentiment analysis in twitter for Macedonian, in: R. Mitkov, G. Angelova, K. Bontcheva (Eds.), Proceedings of the International Conference Recent Advances in Natural Language Processing, INCOMA Ltd., Hissar, Bulgaria, 2015, pp. 249–257. URL: https://aclanthology.org/R15-1034/.

